# Supplementary material for: Mechanisms governing the pioneering and redistribution capabilities of the non-classical pioneer PU.1
Source: Nat Commun. 2020 Jan 21;11:402. doi: 10.1038/s41467-019-13960-2 (PMC6972792; doi:10.1038/s41467-019-13960-2)
Supplement: Supplementary file 7 — Source data [file 41467_2019_13960_MOESM7_ESM.zip › Source_Data/Figure6/Figure6B_MotifScanOutput/ETS-specific/homerResults/motif3.similar.html]

motif3

## Information for motif3

G
A
T
C
A
C
T
G
A
T
C
G
C
G
T
A
C
G
A
T
C
T
A
G
G
C
A
T
T
A
C
G
T
C
A
G
  
Reverse Opposite:  

A
G
T
C
A
T
G
C
C
G
T
A
A
G
T
C
C
G
T
A
C
G
A
T
T
A
G
C
T
G
A
C
C
T
A
G
  

|  |  |
| --- | --- |
| p-value: | 1e-360 |
| log p-value: | -8.308e+02 |
| Information Content per bp: | 1.748 |
| Number of Target Sequences with motif | 957.0 |
| Percentage of Target Sequences with motif | 12.50% |
| Number of Background Sequences with motif | 649.3 |
| Percentage of Background Sequences with motif | 1.61% |
| Average Position of motif in Targets | 99.6 +/- 43.9bp |
| Average Position of motif in Background | 104.6 +/- 57.4bp |
| Strand Bias (log2 ratio + to - strand density) | -0.3 |
| Multiplicity (# of sites on avg that occur together) | 1.01 |
| Motif File: | file (matrix) reverse opposite |

### Similar de novo motifs found

|  |  |  |  |  |  |  |  |
| --- | --- | --- | --- | --- | --- | --- | --- |
| Rank | Match Score | Redundant Motif | P-value | log P-value | % of Targets | % of Background | Motif file |
| 1 | 0.776 | T G C A T C G A T G A C C G T A C T A G T A C G T G C A C A G T A T C G C G A T C A T G C T A G | 1e-187 | -432.869859 | 12.78% | 3.67% | motif file (matrix) |
| 2 | 0.893 | A C G T T A C G A G T C A T G C C T G A A G T C C T G A A C G T G T A C G T A C G A T C T G A C | 1e-117 | -271.686774 | 4.14% | 0.52% | motif file (matrix) |
| 3 | 0.756 | A G T C G A C T A G C T C G A T T A C G C G T A C G A T T A C G G C A T C A T G C A T G A G T C | 1e-111 | -255.748344 | 4.87% | 0.86% | motif file (matrix) |
| 4 | 0.764 | T A G C G A C T A G C T C G A T A T C G C T G A C G A T C T A G G C A T C T A G C A T G | 1e-74 | -172.600595 | 3.71% | 0.76% | motif file (matrix) |
